# Insulin:Glucagon Bipolar Axis in Obesity With a Glimpse Into Its Association With Insulin Resistance in Different Glucose Tolerance States

**DOI:** 10.7759/cureus.58942

**Published:** 2024-04-24

**Authors:** John A Lyngdoh, Happy Chutia, Shanthosh Priyan Sundaram, Vijaya Lakshmi, Alice Ruram, K G Lynrah

**Affiliations:** 1 Physiology, North Eastern Indira Gandhi Regional Institute of Health and Medical Sciences, Shillong, IND; 2 Biochemistry, North Eastern Indira Gandhi Regional Institute of Health and Medical Sciences, Shillong, IND; 3 Community Medicine, North Eastern Indira Gandhi Regional Institute of Health and Medical Sciences, Shillong, IND; 4 General Medicine, North Eastern Indira Gandhi Regional Institute of Health and Medical Sciences, Shillong, IND

**Keywords:** insulin:glucagon ratio, obesity, homa-ir, insulin resistance, hyperglucagonemia

## Abstract

Background: Dysregulation of insulin and glucagon secretion alters the normal insulin:glucagon ratio (IGR) in type 2 diabetes mellitus, obesity, and metabolic syndrome. This study explores the scope of construing the role of these two diametrically opposing hormones on the glucose level not just in obesity but in different glucose tolerance states by looking at the hormone levels and at the insulin glucagon bipolar axis itself.

Materials and methods: This is an analytical cross-sectional study of 60 healthy adults consisting of an equal number of adults who are lean and adults who are obese. It was conducted at North Eastern Indira Gandhi Regional Institute of Health and Medical Sciences (NEIGRIHMS), located in Shillong City, Meghalaya, India. Fasting glucose, insulin, glucagon, and lipids were estimated. Postprandial estimation of glucose was done two hours after oral administration of 75 grams of glucose solution.

Result: The study demonstrated a state of hyperinsulinemia and hyperglucagonemia prevailing in obesity and all sub-categories of the group of persons who are obese. The study showed a higher fasting IGR in the group consisting of adults who were obese (with a mean of 4.11) when compared with the group of adults who are lean (with a mean of 2.24). Fasting IGR was seen to increase with increasing levels of insulin resistance and increasing impairment in glucose tolerance. IGR showed a positive correlation with the homeostatic model assessment for insulin resistance (HOMA-IR) in the impaired fasting glucose (IFG) category and strongly in the impaired glucose tolerance (IGT) category.

Conclusion: Hyperglucagonemia in the group of adult persons who are obese indicates a decreased sensitivity of alpha cells to insulin failing insulin to adequately suppress the secretion of glucagon. The study also demonstrated a positive correlation between IGR and HOMA-IR in obesity and all glucose tolerance states of the group of adults who are obese. It is telltale that the sturdier the insulin resistance, the higher the IGR.

## Introduction

A balanced and diametrically opposite action of insulin and glucagon is essential for the maintenance of blood glucose levels at a range that ensures a continuous adequate supply of glucose to the brain. Conditions like type 2 diabetes mellitus, obesity, and metabolic syndrome are known to be caused by the derangement in the normal plasma concentration of these two pancreatic hormones, which affects the normal balance of glucose [1-3]. Abnormal physiological state like obesity is known to precede the derangement of glucose homeostasis eventually leading to metabolic disorders. The insulin-glucagon bipolar axis represented in the form of the insulin:glucagon ratio (IGR) is therefore an interesting variable to study in people with obesity and with different glucose tolerance states. Although there is a long history of research on the dynamics of secretion and action of these two hormones, there are few studies available in the literature that focus on the bipolar axis of these hormones in different physiological and pathological states. Recently, we have seen a study that proposes the application of IGR to determine the right choice and impact of drugs for managing diabetes [4,5]. The objective of this study is to observe how the alteration of IGR impacts glucose level and tolerance in obesity and to determine the association between fasting IGR and insulin resistance in different glucose tolerance states in adults who are obese and leading a sedentary lifestyle.

## Materials and methods

This cross-sectional and analytical study was given ethical clearance by the Institutional Ethics Committee of North Eastern Indira Gandhi Regional Institute of Health and Medical Sciences (NEIGRIHMS)(NEIGR/IEC/2015/0041). The sample size was a total of 60 adults, which included 30 adults with BMI≥30 kg/m^2^ (persons with obesity) and 30 adults with BMI≤24.9 kg/m^2^ (control group). BMI was calculated as per the WHO formula: BMI ((kg/m^2^) = person's weight (Kg) / square of the person's height (m). The participants were in the age group between 18 and 60 years. The sample size was calculated using the OpenEP software and based on the prevalence of insulinemia among persons with obesity (38%) and persons with no obesity (6%) while assuming a confidence level of 95 % and power of study at 80%. Participants with obesity who were treated for diabetes or diagnosed with disorders that may affect insulin and glucagon secretion were excluded from the study. The groups of persons who were lean (control) and with obesity comprised participants who were employees of a tertiary care center in the Northeast Region of India, including their relatives and friends. Informed consent was obtained from each participant after they had read the patient information sheet written in the language best understood by them.

A consecutive random sampling method was applied to select participants who fulfilled the inclusion criteria. Selected volunteers were asked to come fasting (eight hours overnight) to the department where anthropometric data, such as age, sex, weight, height, waist circumference, and hip circumference, were measured. Venous blood was collected in a plain vacutainer to estimate the fasting values of glucose, lipids, insulin, and glucagon. A 75 g glucose load in 200 ml of water was administered to the participants, and two hours later, a 2 ml blood sample for post-prandial values of glucose, lipids, insulin, and glucagon was again taken. Fasting and post-prandial blood sugar and lipid profile were estimated in a Vitros 4600 automated dry chemistry analyzer (Johnsons and Johnsons, India) using Johnson and Johnson's reagent. Glycosylated hemoglobin was estimated in the Bio-Rad D10 high-performance liquid chromatography (HPLC) machine (Bio-Rad, USA). Fasting and post-prandial plasma insulin were measured in Access II Chemiluminescence using Beckman Coulter reagent (Beckman Coulter, USA). Plasma glucagon was estimated in a Mago 4 automated ELISA processor from Transasia using the ELISA kit. Third-party quality control was done by using the quality control kits from Bio-Rad (USA). Insulin resistance was determined by calculating the HOMA-IR, which is derived by using the following formula:

HOMA-IR = {fasting glucose (mg/dl) x fasting insulin (mU/L)}/405

The study sample was broadly divided into the categories of adults who were lean and obese. The group of persons who were obese was further subcategorized into the normal glucose tolerance group (NGT), the impaired fasting glucose group (IFG), and lastly the impaired glucose tolerance group (IGT) by taking into account the World Health Organization (WHO) criteria for oral glucose tolerance test (OGTT) and data (Consensus statement) from Nathan et al. (2007) [6,7].

Statistical analysis 

Data are presented as mean with standard deviations (SDs) for all variables, which in the case of this study are normally distributive. Pearson’s correlation was applied to correlate variables, like IGR and HOMA-IR, to find their association.

## Results

Anthropometric characteristics

The data for the anthropometric characteristics of the study population include persons who were lean and obese (overall group) and the subcategories of persons with obesity with different glucose tolerance states (Table 1).

**Table 1:** Anthropometric characteristics of the group of persons who are lean, the group of persons who are obese (overall group), and the sub-categories of persons who are obese with different glucose tolerance states. BMI: body mass Index, W/H ratio: waist/hip ratio, NGT: normal glucose tolerance, IFG: impaired fasting glucose, IGT: impaired glucose tolerance. Values are expressed as mean ± SD.

Variable	Control group (n = 30)	Persons with obesity (overall) (n = 30)	Persons with obesity (NGT) (n = 13)	Persons with obesity (IFG) (n = 13)	Persons with obesity (IGT) (n = 4)
Age (years)	31.5 ± 6.15	36.86 ± 8.78	38.30 ± 6.98	36.07 ± 9.97	34.75 ± 11.61
Weight (kg)	47.4 ± 9.05	72.13 ± 22.46	84.30 ± 15.72	79 ± 8.34	84.5 ± 12.39
Height (cm)	149.1 ± 21.24	158.06 ± 9.38	159.69 ± 10.33	156.61 ± 8.24	157.5 ± 11.38
BMI (kg/m^2^)	20.30 ± 2.37	32.51 ± 2.11	32.77 ± 2.57	32.20 ± 1.45	32.75 ± 2.44
Waist (cm)	76.17 ± 9.1	105.1 ± 8.63	107.5 ± 10.18	102.19 ± 3.59	110 ± 10.09
Hips (cm)	84.8 ± 6.64	106.46 ± 5.83	107.23 ± 6.52	104.53 ± 4.74	108.5 ± 8.22
W/H ratio*	0.89 ± 0.06	0.98 ± 0.07	1.00 ± 0.06	0.97 ± 0.05	±0.07

Biochemical estimation

Table 2 shows the higher values of fasting blood sugar (FBS), post-prandial blood glucose (PPBS), and plasma lipid levels in the group of persons who were obese than in the group of persons who were lean.

**Table 2:** Biochemical estimations of the study population categorized into different glucose tolerance status FBS: fasting blood sugar, PPBS: post-prandial blood sugar, TG: triglyceride, Chol: cholesterol, HDL: high-density lipoprotein, LDL: low-density lipoprotein. Values are presented as mean and SD.

Variable	Control group (n = 30)		Persons with obesity (overall) (n = 30)		Persons with obesity (NGT) (n = 13)		Persons with obesity (IFG) (n = 13)		Persons with obesity (IGT) (n = 4)		Total of persons who are lean and with obesity (n = 60)	
	Mean	SD	Mean	SD	Mean	SD	Mean	SD	Mean	SD	Mean	SD
FBS (mg/dl)	91.83	9.15	105.83	17.87	91.92	4.59	113.53	18.43	126	2.82	98.83	15.75
PPBS (mg/dl)	95.16	23.85	132.53	55.69	107.38	19.40	120.69	34.29	252.75	33.92	113.85	46.46
TG(mg/dl)	140.06	82.34	178.96	67.37	160.53	39.60	207	86.84	147.75	35.42	159.51	77.12
Chol (mg/dl)	161.06	37.86	208.63	44.61	218.46	34.64	207.07	51.57	181.75	49.05	184.85	47.52
HDL (mg/dl)	53.23	20.74	47.92	24.64	54	30.17	40.42	12.24	50.42	33.11	50.15	22.88
LDL (mg/dl)	86.89	33.73	130.54	31.24	139.37	15.01	127.68	42.80	120.1	32.69	112.23	38.57

Hormonal values, IGR, and derived variables in different glucose tolerance status

Among the categories of persons who were obese, the group with impaired glucose tolerance (IGT) demonstrated higher biochemical and hormonal mean values than the group with impaired fasting glucose (IFG). However, the p-value by ANOVA is not showing any signicant difference as the group with IGT is too small when compared to the group with IFG. A significant difference in the mean (p<0.05) of all biochemical and hormonal variables was however seen between the control group and all categories with obesity except IGR in IFG and IGT.

The group of persons with obesity (overall) also manifested hyperinsulinemia and hyperglucagonemia in the fasting state when compared with mean values of the control group and when taken our laboratory normal reference range for insulin (2.6-24.9 mIU/ml) and glucagon (50-100 pg/ml) into consideration. The fasting IGR was found to be >3 in the group of persons who were obese and <3 in the group of people who were lean. HOMA-IR is higher in the group consisting of persons who were obese than in the group consisting of persons who were lean (Table 3).

**Table 3:** Hormonal values, insulin:glucagon ratio, and derived variables in different glucose tolerance status FI: fasting insulin, FG: fasting glucagon, IGR: insulin:glucagon ratio, HOMA-IR: homeostatic model assessment for insulin resistance, HbA1c: glycated hemoglobin. Values are represented as mean and SD.

Variable	Control group (n = 30)		Persons with obesity (overall) (n = 30)		Persons with obesity (NGT) (n = 13)		Persons with obesity (IFG) (n = 13)		Persons with obesity (IGT) (n = 4)		Total of persons who are lean and with obesity (n = 60)	
	Mean	SD	Mean	SD	Mean	SD	Mean	SD	Mean	SD	Mean	SD
FI (pmol/l)	16.45	12.92	95.09	66.67	93.33	78.54	84.55	40.68	135.04	95.16	55.77	61.96
FG (pmol/l)	11.08	11.5	28.73	15.24	27.58	20.49	28.3	10.91	33.91	7.01	19.91	16.08
IGR	2.24	1.36	4.11	2.91	5.04	3.90	3.2	1.27	4.03	2.72	3.18	2.44
HOMA -IR	0.62	0.53	4.24	3.16	3.6	3.13	4.03	2.29	7.02	4.93	2.43	2.9
HbA1c	5.57	0.30	6.35	0.4	6.24	0.28	6.24	0.36	7.05	0.05	5.96	0.52

Correlation between HOMA-IR and fasting IGR

**Figure 1:**
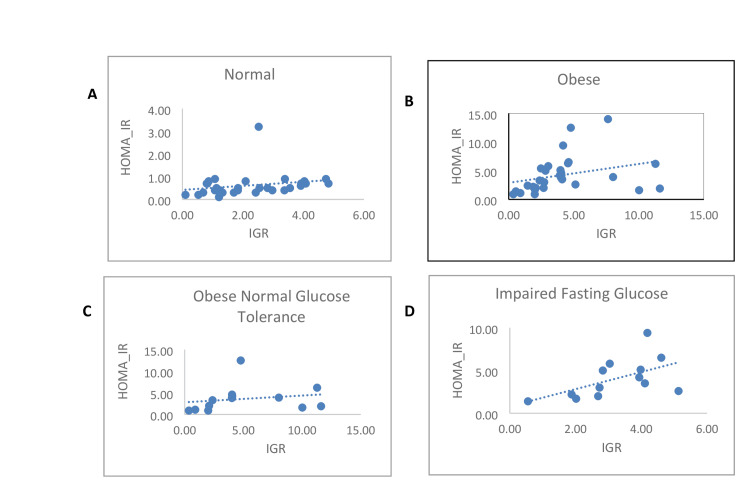
Scatter diagrams depict the correlation between HOMA-IR and fasting IGR in the study population. A: lean population, B: overall obese population, C: obese NGT population, D: obese IFG population

As for the correlation, it was observed that there is a positive correlation between IGR and HOMA-IR in all categories of participants (Figure 1). The correlation between the variables is weakly positive in control (r = 0.26) in the overall participants with obesity (r = 0.3) and in participants with obesity NGT (r = 0.19). The correlation between the variables is strongly positive in participants with obesity IFG (r = 0.55) and in participants with obesity IGT (r = 0.96).

The results also demonstrated a significant difference in the IGR and HOMA-IR values between the category of people who were lean and the categories of people who were obese (overall), the people who were obese with normal glucose tolerance (NGT), the people who were obese with IFG, and the people who were obese with IGT.

## Discussion

The result of this study indicates a higher fasting IGR in the group of persons who are obese compared to the group of persons who are lean (p < 0.05). A similar finding was observed when we investigated insulin resistance, which is determined by the derivation of HOMA-IR, whereby the group of persons who are obese demonstrates higher HOMA-IR (with a mean of 4.2) than the group of persons who are lean (with a mean of 0.62). This strongly indicates that insulin resistance exists in the category of persons who are obese who participated in this study. To correlate fasting IGR and insulin resistance in different glucose tolerance states, we break the group of persons who were obese into different tolerance states by taking into account the WHO criteria for OGTT and data from Nathan et al. (2007), which then gave us four groups consisting of people who are lean with normal glucose tolerance, people who are obese with NGT, people who are obese with IFG, and people who are obese with IGT. Having done so, we noticed that there is a similar picture emerging in all the subcategories of participants with obesity wherein the fasting IGR mean values in the overall participants with obesity, participants with obesity NGT, IFG, and IGT are higher than in the control group. However, on testing the difference with ANOVA, we saw a significant difference only between the control group and overall participants with obesity (p < 0.05) and with NGT (p < 0.05). While looking at HOMA-IR, it is observed that the mean value increased with increased impairment of glucose tolerance. There is also a significant difference in HOMA-IR value between the control and all categories of obesity with different glucose tolerance states (p < 0.05).

There is a dearth of studies of this nature in which the correlation between IGR and insulin resistance in different glucose tolerance states is highlighted. Only a few classical studies had addressed the importance and the relevance of the IGR in glucose homeostasis, and these were conducted by the discoverer of glucagon himself (Dr. R.H. Unger) and his colleagues who had worked in association with him [8,9]. Their studies revealed that obesity is characterized by increased IGR under arginine stimulation. Similarly, the results of our study observed a high IGR in the category of persons who are obese. The blood glucagon levels seen in the group consisting of persons who are obese in our study are seen to be higher than that of the population who are overweight in another study [9]. There is a state of hyperglucagonemia in the categories of persons who are obese in our study (Table 3), but because of the co-occurrence of hyperinsulinemia, the IGR still lingers within the range that is observed in pancreatic hormonal secretion in the IGT state, which is manifested by the alteration many other studies [8-10,11]. This confirms that there is an anabolic state in obesity, a condition that is driven by insulin, a hormone that is secreted in a greater proportion than glucagon even in a fasting state. Hyperglucagonemia seen in the obese group is a consequence of decreased sensitivity of alpha cells to insulin, resulting in insulin's failure to adequately suppress the secretion of glucagon as described by many workers involved in a similar field of study [12,13]. The hyperglycemia observed in the IFG state and IGT state implicates a bi-hormonal effect in which glucagon raises the hepatic glucose output, and insulin diminishes the peripheral utilization of glucose. This corroborates with suggestions made in some studies, which regarded that the cause of hyperglycemia in the IFG state and IGT state is both insulinotropic and glucagonotropic in nature [14,15]. In-depth studies looking into hormonal content within the pancreas in diabetics have reported findings of the reduction of insulin stores in the pancreas and glucagon stores that remained unchanged [16]. This is a scenario that substantiates the notion that glucagon plays a significant role in the cause of hyperglycemia in diabetics as glucagon stores remain unaffected in diabetics. We can also say that this forms the basis of understanding the prevailing dysregulation in the IGR. 

The findings of our study may appear to be in confluence with earlier studies, but it is to be noted that unlike these earlier studies [8,13], our study did not use arginine as a challenge to stimulate the secretion of pancreatic hormones. Our study however used the standard 75 g glucose load only as a challenge for hormone secretion and to determine the glucose tolerance state. Assessment of glucose tolerance enabled the sub-categorization of the group of persons who are obese into different glucose tolerance states and provided us the opportunity to compare and have a glimpse of the correlation between IGR and insulin resistance in all the different glucose tolerance states. From the overall findings and the findings of the existence of a positive correlation of IGR with HOMA-IR in all the subcategories of the group of people who are obese, we can conclude that a higher insulin-glucagon bipolar axis is an indication of increased insulin resistance. The same may be also stated vice versa, that in the presence of insulin resistance, the IGR is also raised.

The study has a limitation in that the category of people who are overweight is left out of this study and that this study focused only on the population of people who are obese with obesity of grade 1 and above. If data were also available on the category of people who are overweight, then it would have given us a thorough perspective of the association between IGR and insulin resistance in different glucose tolerance states and in the broader spectrum of BMI. Although this study has applied the WHO BMI classification 1998 to subcategorise the study sample, as the study participants of this study are of Asian Indian background, the study is also therefore a representation of the Asian Indian population and perspective. Thus, the inclusion of the overweight category would have been an inclusion of the wider spectrum of people with obesity if the WHO Asian Indian classification of obesity is to be taken into consideration [17]. 

As for biochemical limitations, glucagon suppression by insulin could have been explored and examined comprehensively by extending the scope for estimation of glucagon levels of samples that could be taken every half hour after the 75 g glucose challenge for two hours. Estimation of other insulin and glucagon regulators like somatostatin and incretins could have also been included to give us a wholesome picture of insulin and glucagon secretion in obesity and in different glucose tolerance states. This study therefore provides us a glimpse of the effect of hyperglucagonemia on the blood glucose level and on the presence of a positive correlation between IGR and insulin resistance in the groups of persons who are obese with IFG and IGT.

## Conclusions

From this study, we can conclude that there is a high occurrence of hypergluconemia in adults with obesity. This indicates a decreased sensitivity of alpha cells to insulin failing insulin to adequately suppress the secretion of glucagon. The hyperglycemic state observed in IFG and IGT states implicates a bi-hormonal effect wherein glucagon raises the hepatic glucose output and insulin decreases the peripheral utilization of glucose. In other words, the finding implies that the cause of hyperglycemia in the overall group of participants who are obese and in obese subgroups with different glucose tolerance states is insulinotropic and glucagonotropic in nature. The study also provided us with a glimpse of the existence of a positive correlation between IGR and HOMA-IR in the participants who are obese and in all the subgroups of obese participants with different glucose tolerance states. It is telltale that the higher the IGR, the sturdier the insulin resistance.
